# Colour Variants in Siberian Cats: A Comprehensive Review of Phenotype, Genetics, and Breed Registry Standards

**DOI:** 10.3390/genes17020208

**Published:** 2026-02-09

**Authors:** Agnieszka Górska, Bartłomiej Zieniuk, Marlena Wojciechowska

**Affiliations:** 1 Department of Chemistry, Institute of Food Sciences, Warsaw University of Life Sciences-SGGW, 159C Nowoursynowska Str., 02-776 Warsaw, Poland; bartlomiej_zieniuk@sggw.edu.pl; 2 Department of Animal Genetics and Conservation, Institute of Animal Sciences, Warsaw University of Life Sciences-SGGW, 8 Ciszewskiego Str., 02-786 Warsaw, Poland; marlena_wojciechowska@sggw.edu.pl

**Keywords:** Siberian cat, Neva Masquerade, coat colour genetics, feline genetics

## Abstract

Siberian cats are characterized by a high level of genetic variability, which is also reflected in a wide range of colour variations. Knowledge about the genetic background of these coat colour varieties is fragmented and predominantly derived from research on other breeds, with inconsistencies in nomenclature across major feline organizations. This review aims to offer a comprehensive synthesis of the genetic mechanisms underlying coat colour and pattern variation in Siberian cats, while also critically examining how these phenotypes are defined, named, and recognized across key international feline breed registries. In Siberian cats, as in other breeds, the fundamental factor in the development of a phenotype is the interaction of multiple genes involved in the production of various types of melanin, its quantity, and distribution in the skin and coat. An analysis of breed standards revealed inaccuracies in the naming of several traits and differences in the acceptance of certain phenotypes within the breed, most notably concerning basic colours, ticked patterns, colourpoint recognition, silver and golden variants, as well as definitions of white spotting categories. The Siberian cat exhibits complex and partially breed-specific genetic determinants of coat colouration. Unification of nomenclature among feline federations would improve clarity in breeding practice and genetic documentation. Some of the traits still require molecular research into their genetic background, making the breed interesting not only to cat lovers but also to researchers.

## 1. Introduction

Although records of ancient Siberian cats date back to the 10th century, the official breed can be considered relatively young [1,2,3]. The first breed standard for the Siberian cat was developed in the late 1980s by the felinological club Kotofei. This standard was based on the phenotype of semi-longhaired cats native to the territories of the former USSR and was intended to distinguish the breed from other semi-longhaired cats, such as the Maine Coon and the Norwegian Forest Cat. From its inception, the standard included characteristic features such as a powerful body type, solid bone structure, round paws, a full and rounded muzzle, widely set ears, oval eyes, and a rounded head shape. In 1990, the standard was further refined and officially recognized coat colour categories were introduced, including agouti, agouti with white, non-agouti, non-agouti with white, and colourpoint patterns within the same groups. In 1991, the first international breed standard—also encompassing the Siberian colourpoint variety—was developed and approved by the World Cat Federation (WCF) [1,2,3,4].

To date, the novice class (for cats with unknown parents or without a pedigree, allowing them to be shown once under specific conditions before possible registration into a target breed) remains open for Siberian cats originating from the territories of the former USSR, allowing for the continued introduction of new individuals into the breeding population and thus contributing to the expansion and maintenance of genetic diversity. This approach is exceptional within pedigree cat breeds, as the novice class is closed for nearly all other recognized breeds, making the Siberian cat one of the few breeds for which controlled inclusion of foundation cats is still permitted [1,3].

Analysis of the genetic diversity of this breed revealed that it is among the highest of all popular breeds. The estimated observed heterozygosity was 0.69, compared to an average of 0.51 for all breeds [5]. High genetic variability is also reflected in the low inbreeding coefficient, as well as the low percentage of genetic diseases recorded in representatives of the breed [5,6]. Despite the growing popularity of the breed, the genetic foundations of its diverse coat colours remain insufficiently synthesized across the literature. Coat colour, in particular, represents a visible and highly heritable trait that plays a central role in breed identification, esthetic preferences, and breeding strategies. Existing studies describe individual loci and phenotypes in various breeds; however, no comprehensive review has yet integrated these findings into the standard of Siberian cats. Conflicting terminology, varying classification systems in different organizations, and incomplete genetic data have created uncertainty around the colour varieties of Siberian cats. Accurate knowledge of coat colour genetics is essential for making informed breeding decisions, predicting phenotypes, and maintaining breed standards.

Two primary types of melanin pigments determine coat colour in domestic cats: eumelanin and pheomelanin. Eumelanin is responsible for producing shades ranging from deep black to warm brown, while pheomelanin generates red to cream hues [7].

The relative presence and distribution of these pigments define the visual appearance of a cat’s coat and depend on allelic and non-allelic interactions between different genes. This review aims to synthesize current molecular and classical genetic knowledge about coat colour and pattern variation in Siberian cats. It also critically compares how these phenotypes are defined, named, and recognized by major international feline organizations within the Cat World Congress. Specifically, this review analyses breed standards and colour nomenclature applied by the World Cat Federation (WCF) [1], The International Cat Association (TICA) [2], Fédération Internationale Féline (FIFe) [3], the Australian Cat Federation (ACF) [8], the Co-ordinating Cat Council of Australia (CCCA) [9], Cat Fanciers’ Association (CFA) [10], the Governing Council of the Cat Fancy (GCCF) [11], New Zealand Cat Fancy (NZCF) [12], and the Southern African Cat Council (SACC) [13]. Particular attention is paid to differences in phenotype recognition and classification among these organizations, including cases where colour specifications are not defined for the breed (e.g., in the NZCF, where Siberian cats are registered as SIB without colour descriptions) and situations where no official breed standard is available (e.g., in the CCCA). By combining genetic evidence with registry-based nomenclature, this review seeks to identify inconsistencies, emphasize breed-specific colour variants, and pinpoint areas where terminology needs harmonization and further molecular research is necessary to enhance clarity in breeding practices and genetic documentation.

## 2. Review

### 2.1. Base Colour

#### Loci B and O

The eumelanin pigment in various cat breeds is represented by three colour varieties, black, chocolate, and cinnamon, and their modifications. All of them are determined by variants in tyrosinase-related protein 1 (*TYRP1*), the B locus that modifies eumelanin synthesis [6]. Depending on the registry or source, eumelanin-based pigmentation may be described as black or seal, the latter term being particularly common for colourpoint cats carrying the *c^s^* allele [1,2,3,8,9,10,11,13]. In some cat fancy federations, particularly those based in the United States, the black coat colour is sometimes informally referred to as “brown.” However, official breed standards and colour descriptions published by these organizations consistently identify the correct and formal designation of this colour as black. The Siberian cat breed standard only allows black and its modifications [1,3,8,9,10,11,12,13], with the exception of TICA, which is the only organization that does not explicitly state that chocolate and cinnamon are prohibited [2].

The pheomelanin pigment is referred to as red in feline breeding terminology, although the scientific literature often uses alternative descriptors such as orange, yellow, or ginger. The intrinsic cellular “switch” that controls pigmentation in many species, including cat breeds, is the ASIP-MC1R pathway. Activation of melanocortin-1 receptor (MC1R) by α-melanocyte stimulating hormone (α-MSH) promotes eumelanin production, while agouti signalling protein (ASIP) antagonizes MC1R, leading to pheomelanin production. A receptor dysfunction resulting from a mutation or deletion in its gene can also lead to changes in the type of melanin produced in the cell, creating pheomelanistic phenotypes such as amber or russet. Therefore, the coat colour (black/brown versus red/yellow) largely reflects the state of MC1R signalling, with other genetic loci influencing tone or distribution [14,15,16].

**Figure 1:**
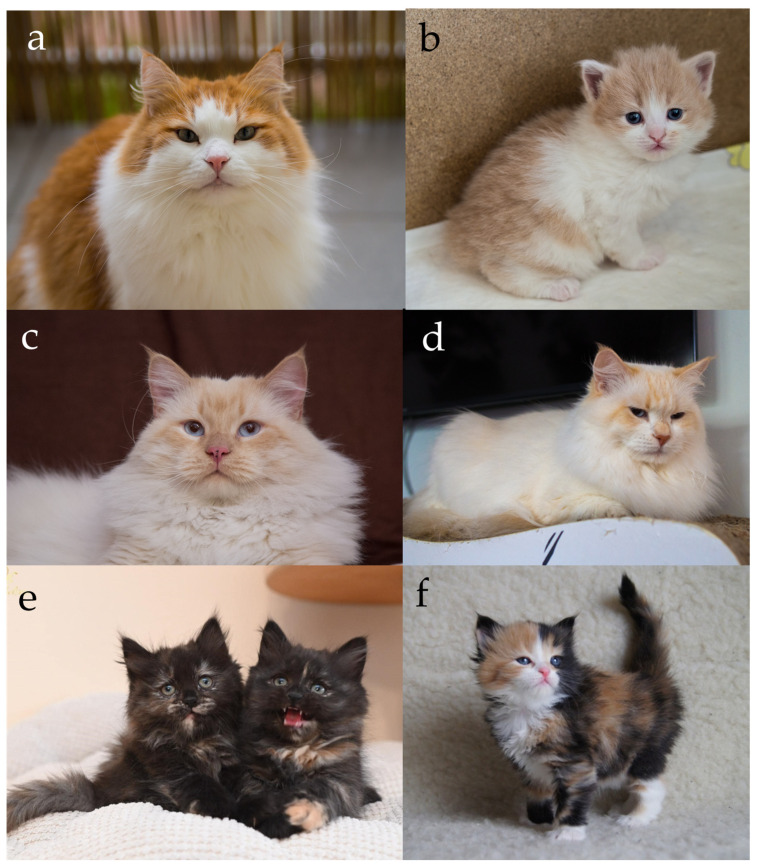
Expression of the red pigments in Siberian cats. (**a**) Red tabby with white, (**b**) cream tabby with white, (**c**) cream point with white (visible coloured chin and ears edges and dark spots on the nose that are typical of non-agouti cats), (**d**) red tabby point with white, (**e**) two black tortie sisters without white (visible mixed red and black hair), (**f**) black tortie with white (visible larger spots of red and black). Red cats have a pink nose leather. Source of photographs: (**a**–**d**) cattery First Snow, (**e**) cattery Blue Chelsea, (**f**) cattery od Ivanki.

In the case of Siberian cats, only one allele version of *MC1R* is present [6], so the genetic basis for the switch between eumelanin and pheomelanin production lies in the *O* locus, located on the X chromosome. The *O* allele, commonly known as the Red gene, converts eumelanin synthesis into pheomelanin, resulting in red-based pigmentation. Because the gene is sex-linked, its inheritance follows a pattern dependent on the cat’s sex. Males, being hemizygous (XY), express either red or non-red pigmentation depending on whether they inherit the *O* or *O*’ allele (Figure 1a,b). Females (XX), however, may be homozygous or heterozygous. Heterozygous females (*O*/*O*’) exhibit both pigment types simultaneously, resulting in the tortoiseshell (tortie) phenotype caused by random X-chromosome inactivation (Lyonization) during early embryonic development [7,17,18,19,20].

In red cats, whether tabby or non-agouti, the coat frequently displays a visible pattern of faint striping. This occurs because the pheomelanin pigment has a lower contrast in hair shaft banding than eumelanin, making it difficult to distinguish truly non-agouti individuals from tabby ones. Even red cats without the agouti allele often exhibit “ghost tabby” markings, residual stripes that remain visible on the body [21]. The distinction between agouti and non-agouti red cats can sometimes be observed through subtle details: the edge of the ear tends to be paler, the chin is usually lighter in tone, and self-coloured red cats often have small freckles or dark spots on the nose leather or lips (Figure 1c,d). These characteristics make complete visual differentiation challenging, particularly in young individuals.

In tortoiseshell females, the appearance of the two pigments depends on the presence or absence of white spotting. In cats without white, the transition between the black and red areas often is gradual, producing a mottled or intermingled pattern where both pigments appear finely mixed throughout the coat. In contrast, tortoiseshell females carrying white spotting usually display distinct and clearly separated patches of black and red. The white patterning provides natural borders that enhance the contrast between these two colours, making the mosaic appearance more defined and visually striking (Figure 1e,f) [20]. A similar relationship is observed in agouti cats, where the presence or absence of white spotting likewise influences the sharpness of colour boundaries; moreover, within the coloured patches, the underlying tabby striping remains visible, revealing the agouti pattern expressed locally within each pigment area.

The molecular basis of red pigmentation in domestic cats has been clarified through detailed genomic studies. Two independent investigations identified a ~5 kb deletion within an intronic region of the Rho GTPase Activating Protein 36 (*ARHGAP36*) gene on the X chromosome, corresponding to the O locus. This deletion leads to ectopic, melanocyte specific expression of *ARHGAP36*, an Rho GTPase-activating protein that is not otherwise implicated in pigmentation in mammals. Expression of *ARHGAP36* in melanocytes reduces the levels of the catalytic subunit of protein kinase A (PKA), thereby suppressing the activity of melanogenic genes downstream of the melanocortin 1 receptor signalling pathway. Although MC1R itself remains functional, pigment synthesis shifts from eumelanin toward pheomelanin, producing the characteristic red or yellow hair colour associated with the Red locus [22,23].

From a breed-standard perspective, both the red colour and its diluted form, known as cream, are recognized and accepted by all major feline federations. In most feline organizations, females expressing both eumelanin (black) and phaeomelanin (red) pigmentation are collectively referred to as “tortie”. However, in the official CFA nomenclature, the terminology is more specific: a non-agouti female is designated “tortoiseshell”, an agouti female is termed “patched”, and the presence of white spotting results in the designation “calico”. Within the same registry, red-pointed cats are referred to as “flame”, while red silver individuals are termed “cameo” [10]. In the NZCF system, the term “black” is generally omitted from colour descriptions and is retained only for colourpoint cats, which are designated as “seal”; nevertheless, NZCF uniquely applies the single term “black” to describe black tortie females and uses “bluecream” to denote blue tortie individuals [12]. Additionally, informal terminology encountered internationally includes the use of “torbie” for tortie-tabby females and “tricolour” for tortie females exhibiting white spotting.

### 2.2. Modifiers

#### 2.2.1. Locus A

As mentioned previously, the cellular “switch” that determines whether melanocytes produce eumelanin or pheomelanin is primarily regulated by interactions between MC1R and its ligands. When MC1R is active, either through its constitutive activity or by binding of agonists such as α-MSH or adrenocorticotrophic hormone (ACTH), a Gs protein- and adenylate cyclase-dependent signalling cascade is initiated. This results in increased intracellular cyclic adenosine monophosphate (cAMP) concentration, activation of protein kinase A (PKA), and phosphorylation of CRE-binding protein (CREB) transcription factors in the nucleus. Consequently, microphthalmia-associated transcription factor (MITF) expression is upregulated, leading to an increase in the expression of melanogenic enzyme genes, including tyrosinase (*TYR*) and tyrosinase-related proteins, *TYRP1*, and *TYRP2*, thereby directing the cell toward eumelanin synthesis.

Agouti-driven coloration in cats results from the timing and location of melanocyte signalling at the *A* locus, which encodes agouti signalling protein. ASIP is a small, secreted molecule that antagonizes the melanocortin 1 receptor on melanocytes. ASIP, by binding to the receptor, suppresses its basic activity, and the C-terminus of ASIP blocks the binding side of α-MSH. This leads to the inhibition of the cAMP–PKA–MITF pathway and a decrease in tyrosinase activity. Melanocytes exhibiting low tyrosinase activity tend to synthesize pheomelanin in the presence of cysteine, resulting in the formation of a yellow band in agouti hair [24].

**Figure 2:**
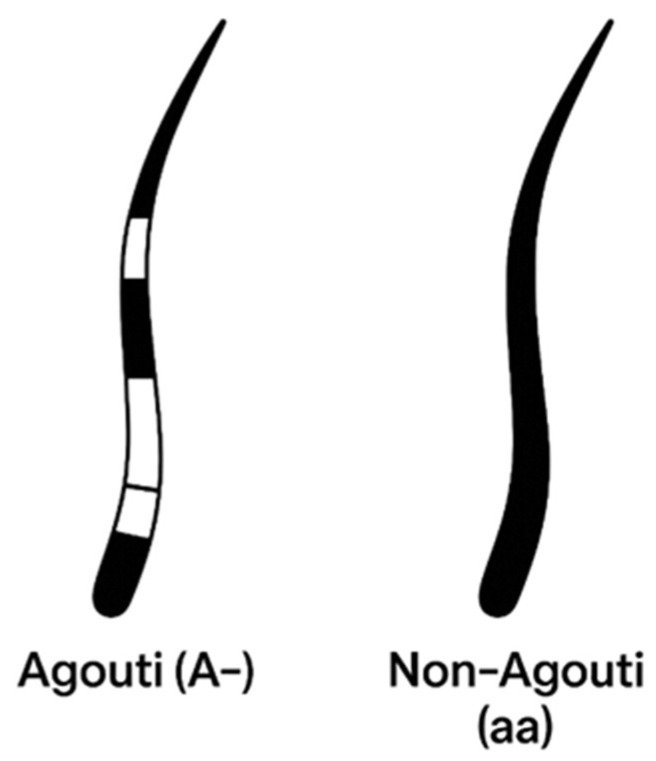
Agouti and non-agouti cat hairs.

**Figure 3:**
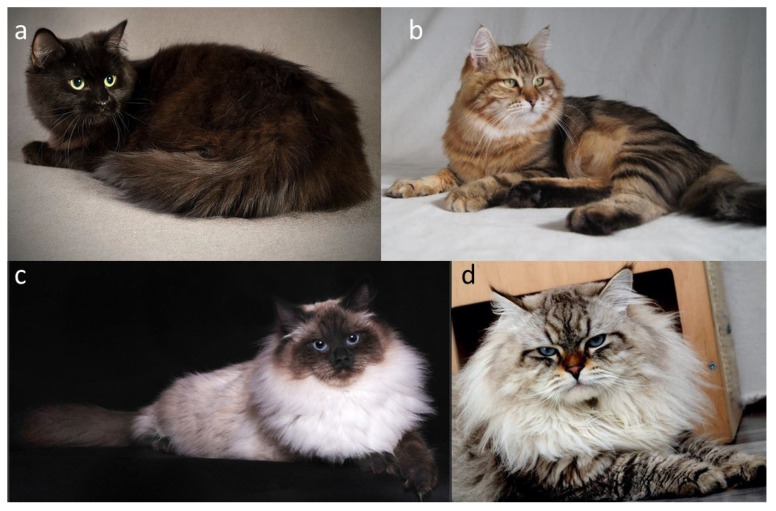
Agouti and non-agouti Siberian Cats. (**a**) Black (non-agouti), (**b**) black tabby (agouti), (**c**) seal point (expression of non-agouti in colourpoint), (**d**) seal tabby point (expression of agouti in colourpoint). Agouti cats typically exhibit a characteristic “M”-shaped marking on the forehead, which is a well-recognized phenotypic feature associated with agouti patterning. It is worth mentioning that black agouti cats have a brick nasal leather with a presence of dark edges, while non-agouti cats have a color that matches the color of their fur. Additionally, agouti cats typically exhibit a white or pale chin, which is a characteristic feature of the agouti pattern and should not be interpreted as evidence of the presence of the white spotting gene. Source of photographs: (**a**,**b**) cattery od Ivanki, (**c**,**d**) cattery Błękitny Anioł.

The cyclical on–off expression of *ASIP* during the hair growth cycle creates banded (agouti) hairs, with alternating dark eumelanin and light pheomelanin segments along the shaft (Figure 2 and Figure 3). Regional differences in *ASIP* expression across the skin establish the characteristic agouti background on which other patterning genes act [25,26,27,28].

In domestic cats, the classic agouti vs. non-agouti distinction reflects allelic variation at the *ASIP* locus. The wild-type agouti allele (*A*) encodes a full-length, functional ASIP that is expressed in a temporally pulsed fashion in hair follicles, yielding hairs with distinct light and dark bands and a visibly tabby or ticked background (Figure 3b,d). By contrast, the common non-agouti allele (*a*) is caused by a 2 bp deletion in exon 2 of *ASIP* that introduces a frameshift and truncates the C-terminal signalling domain [29]. This loss-of-function allele prevents ASIP from effectively antagonizing MC1R, resulting in melanocytes remaining in a state of eumelanin production throughout the hair cycle. Cats that are homozygous *a*/*a* therefore lack banding and appear solid black (or their dilute equivalents) (Figure 3a,c), with any underlying pattern only faintly visible as “ghost” tabby in some individuals. This molecular lesion provides the mechanistic basis for the long-recognized recessive non-agouti phenotype at Locus *A*.

Historically, the agouti locus (*A*) was defined in classical coat-colour genetics as the determinant of “agouti tabby versus self (solid)” phenotypes long before its molecular identity as ASIP was known. Subsequent mapping and cloning studies in domestic and wild felids confirmed that this locus corresponds to ASIP. The domestic non-agouti phenotype is specifically linked to the exon-2 frameshift deletion, while melanism in several wild cat species results from different mutations in other pigmentation genes, such as *MC1R* [29]. Comparative research across mammals has shown that *ASIP* acts as a conserved switch regulating the balance between eumelanin and pheomelanin, with recurrent loss-of-function alleles at the agouti locus causing melanism in many lineages [27,28,29].

From a practical standpoint, variation at Locus *A* is now routinely assayed in feline genetic testing. Commercial veterinary laboratories genotype the domestic cat *ASIP* frameshift using PCR-based assays (often allele-specific PCR or TaqMan indel assays) on DNA from buccal swabs or blood, and report results as *A*/*A* (agouti), *A*/*a* (agouti, carrier of *a*), or *a*/*a* (non-agouti) [30,31]. These tests allow breeders and researchers to infer the underlying *A*-locus genotype from the visible coat and to separate true non-agouti homozygotes from cats whose pattern may be modified or masked by other loci that will be discussed in subsequent sections.

All cat organizations recognize the agouti and non-agouti patterns in the Siberian breed, and in coat colour nomenclature, agouti Siberian cats are described using the term “tabby”.

#### 2.2.2. Locus Mc (Ta), Sp, Ta (Ti)—Modifiers of Tabby Pattern

**Figure 4:**
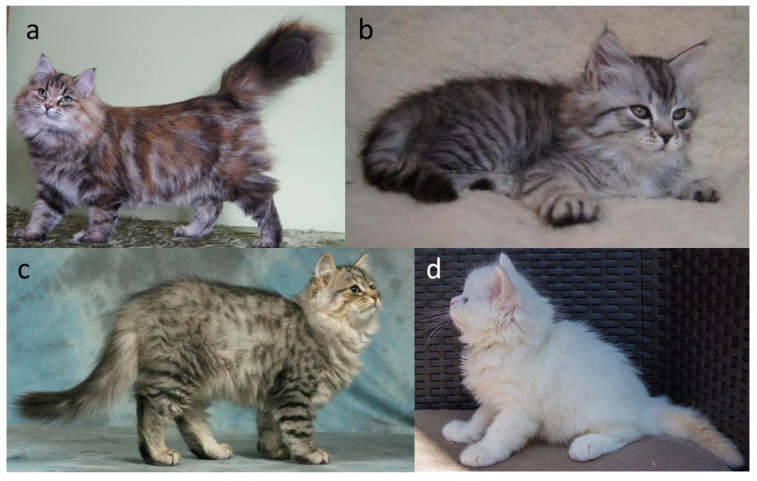
Tabby patterns in Siberian cats. (**a**) Black tortie classic (blotched) tabby, (**b**) black silver mackerel tabby, (**c**) black silver spotted tabby, (**d**) cream tabby point—this is an agouti cat, but he is very light in colour and it is impossible to assess the tabby pattern (some cat’s organizations use term “unspecified tabby pattern”). In young Siberian cats, the tabby pattern is relatively easy to identify; however, in adult individuals the coat becomes longer and denser, which may significantly hinder accurate determination of the pattern. Source of photographs: (**a**–**c**) cattery od Ivanki, (**d**) cattery First Snow.

Tabby markings in domestic cats are produced by the interaction of several loci that control how a dark pattern is laid down on an agouti background. In practice, we distinguish four main types of tabby pattern: blotched (classic) (Figure 4a), mackerel (Figure 4b), spotted (Figure 4c), and ticked (no pictures for ticked Siberians). These pattern classes are conserved across many breeds and closely parallel the range of stripes, whorls, and spots seen in wild felids [25,26].

Before molecular genetics elucidated the mechanisms underlying the inheritance of tabby coat patterns, numerous hypotheses were proposed to explain the transmission of striping phenotypes. These theories were largely derived from systematic breeding observations and phenotypic analyses conducted by early geneticists and breeders. At present, the most widely accepted classical model assumes that the blotched (classic) tabby pattern is recessive to the mackerel pattern, with both phenotypes being controlled by the *Mc* locus. In mackerel cats, the dark coat components form narrow, evenly spaced vertical stripes; in blotched (classic) cats, the same dark areas expand into broad whorls with a “bull’s-eye” on the flank [25]. According to classical genetics, cats that are *Mc*/– display the mackerel pattern, while *mc*/*mc* homozygotes show the classic/blotched pattern. The spotted pattern acts as a modifier of *Mc* and is usually linked to a separate locus called *Sp*. In the simplest traditional model, cats that are sp/sp have no spotting effect and show the pattern determined by *Mc*: *Mc*/–, *sp*/*sp* cats are mackerel, while *mc*/*mc*, *sp*/*sp* cats are blotched. In contrast, cats with at least one *Sp* allele (*Sp*/*Sp* or *Sp*/*sp*) display a spotted tabby pattern because the continuous mackerel or blotched bars are broken into spots or rosettes along the sides [32,33]. A distinct tabby phenotype is represented by the ticked pattern, which is controlled by the *Ta* (ticked agouti) locus. The presence of the *Ta* allele results in a strong reduction or complete suppression of visible tabby markings on the trunk. Cats expressing the ticked phenotype exhibit an overall uniform appearance of the coat, caused by fine, alternating bands of eumelanin and pheomelanin along individual hairs. As a consequence, the coat lacks clearly defined stripes or whorls, although residual markings may still be observed on the extremities, tail rings, or facial patterning.

Advances in molecular genetics have verified and refined earlier assumptions derived from classical breeding studies concerning the inheritance of tabby coat patterns in domestic cats [25,27,34]. These studies demonstrated that variation at a single major genetic locus underlies the phenotypic distinction between the mackerel and blotched (classic) tabby patterns. Specifically, the gene Transmembrane aminopeptidase Q (*Taqpep*, also known as *LVRN*) was identified as the primary determinant of stripe periodicity and pattern organization, thereby explaining the morphological differences observed between these two tabby phenotypes. In addition, Endothelin 3 (Edn3) was identified as a key regulator of pigmentary differentiation within individual hairs. Edn3 exerts a significant influence on localized melanin production by melanocytes, contributing to the contrast between dark and light regions of the coat. Based on combined genetic, histological, and gene expression analyses, the authors proposed a two-stage model of tabby pattern formation. In the first stage, *Taqpep* establishes a spatial “pre-pattern” in the developing skin, defining the future arrangement of dark and light regions. In the second stage, differential expression of *Edn3* stabilizes and reinforces these pre-established domains by modulating melanocyte activity and pigment synthesis within hair follicles. Histological examinations and gene expression studies, including analyses performed in transgenic model systems, indicate that *Edn3* is expressed in a paracrine manner within the dermal papillae of hair follicles, where it coordinates localized differences in hair pigmentation. In their genetic model, the authors designated allelic variation at the *Taqpep* locus as *Ta^M^* for the mackerel pattern and *Ta^b^* for the blotched pattern, a nomenclature corresponding to the traditionally used *Mc* and *mc* alleles in classical genetic descriptions.

Importantly, molecular genetic analyses have not confirmed the existence of an independent *Sp* locus as postulated in earlier classical models. Despite the phenotypic distinction between continuous striped and spotted tabby patterns, no separate genetic locus responsible solely for spotting has been conclusively identified to date. Instead, current evidence indicates that cats exhibiting a broken or spotted tabby pattern are, from a genetic perspective, also mackerel-patterned cats [25,26]. In these individuals, the characteristic vertical stripes of the mackerel pattern are fragmented into discrete spots or short segments along the flanks, without a change in the underlying *Taqpep* genotype associated with stripe periodicity. This observation supports the view that the spotted phenotype represents a modification of the mackerel pattern rather than a distinct pattern category controlled by a separate locus. Consequently, the classical concept of the *Sp* locus is now considered unsupported by molecular data, and spotted tabby cats are best interpreted as a phenotypic variant within the mackerel pattern continuum.

Earlier classical models proposed that ticked was part of an allelic series at the tabby locus that also included the mackerel and blotched alleles (*Ta^M^* and *Ta^b^*, respectively). However, subsequent molecular mapping demonstrated that the ticked phenotype is not allelic to *Taqpep* but instead corresponds to an independent locus located on chromosome B1 [29,32,34]. Developmental analyses have shown that the ticked phenotype is associated with altered expression of Dickkopf WNT Signalling Pathway Inhibitor 4 (*DKK4*), a key regulator of WNT signalling during skin and hair follicle development [26]. In tabby-patterned cats, periodic expression of *DKK4* in the embryonic epidermis establishes alternating domains of high and low signalling activity, which correspond to future dark and light regions of the coat. In contrast, cats carrying the ticked-associated allele exhibit a disruption of this spatial periodicity, resulting in a more uniform epidermal signalling landscape during development. As a consequence of this altered *DKK4*-mediated signalling, the pre-pattern that normally gives rise to distinct stripes or whorls fails to resolve into macroscopic tabby markings. Although the underlying developmental framework for pattern formation is established, the suppression or homogenization of *DKK4* expression prevents the amplification of contrast between adjacent regions. This leads to the characteristic ticked appearance, in which individual hairs retain agouti banding but organized tabby markings on the trunk are reduced or absent, with residual patterning often confined to the extremities. Importantly, the *Ti^A^* allele corresponds to the earlier classical designation *Ta* used in traditional cat coat colour genetics.

Because these patterns appear on individual hairs and can be affected by hair length, practically identifying the pattern requires considering age and coat type. Like other tabby variants, the underlying pattern is easiest to recognize in early kittenhood, when the coat is short and the contrast between dark and light areas is sharp. It tends to become less distinct as the coat thickens or lengthens. Across major feline organizations, the nomenclature and acceptance of tabby patterns in Siberian cats show notable variation. Most registries recognize the classic (blotched), mackerel, spotted, and ticked patterns; however, the GCCF is an exception, as it does not permit the ticked pattern in the Siberian breed [10]. Although most organizations’ standards allow the ticked tabby pattern, cats with this pattern are not common. In contrast, both TICA and SACC additionally accept the marbled pattern, expanding the range of permissible tabby expressions [2,13]. In TICA and CFA, colourpoint cats exhibiting tabby markings are referred to as “lynx”, a terminology widely used in North American registries [2,10]. In some cases, determining the exact tabby pattern is not feasible, either due to extensive white spotting or because the colourpoint phenotype inherently obscures pattern visibility. Under such circumstances, several organizations designate the pattern simply as “tabby”, indicating an unspecified tabby pattern, especially for the colourpoint cats [1,2,3,8,9,10,11,12,13].

#### 2.2.3. Locus C

The *C* locus plays a fundamental role in determining coat pigmentation in domestic cats. The wild-type allele, designated as *C*, encodes a fully functional tyrosinase enzyme that enables the complete synthesis of eumelanin (black pigment) and pheomelanin (red pigment), depending on the allelic composition at other loci involved in pigment regulation. Cats carrying this allele express uniform, fully pigmented coats without regional colour restriction (for example Figure 1a,e, Figure 3a,b and Figure 4a–c) [7].

**Figure 5:**
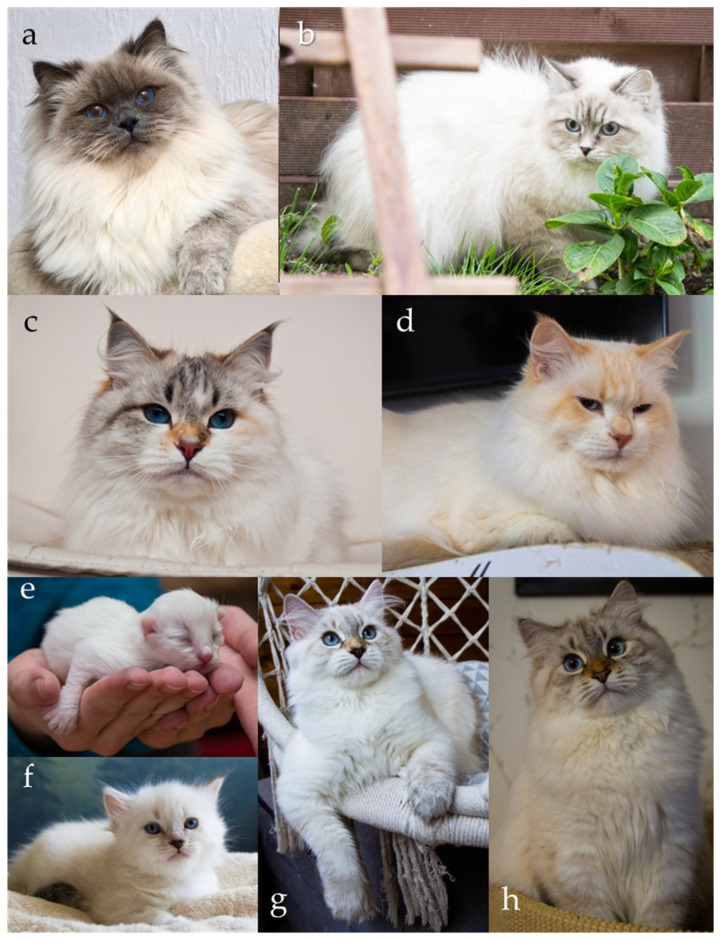
Colourpoint Siberians (Neva Masquerade). (**a**) Blue point, (**b**) blue tabby point, (**c**) seal tortie tabby point with white, (**d**) red tabby point with white. Photos (**e**–**h**) show colourpoint development in the same cat at different ages: (**e**) one day old, (**f**) 5 weeks old, (**g**) 14 weeks old, (**h**) 7 months old. Source of photographs: (**a**) cattery Błękitny Anioł, (**b**–**h**) cattery First Snow.

A key variant of the tyrosinase (*TYR*) gene within the C locus is the autosomal recessive *c^s^* allele, responsible for the colourpoint phenotype, also known as point, oculocutaneous albinism, acromelanism, Siamese, or the Himalayan coat-colour pattern (Figure 5a–h). This allele produces a temperature-sensitive form of tyrosinase, an enzyme essential for melanin synthesis. The altered tyrosinase becomes unstable and inactive at normal body temperature, which suppresses pigment production in warmer regions of the body, such as the torso. In contrast, the enzyme remains active in cooler, distal regions, including the face, ears, paws, and tail, resulting in the characteristic pattern of darker “points” contrasting with a lighter body. Kittens homozygous for the *c^s^*/*c^s^* genotype are typically born nearly white (Figure 5e), as they are evenly warmed within the maternal uterus and the enzymatic activity of tyrosinase remains inhibited during fetal development. As the kittens age and their body temperature distribution changes, pigmentation gradually appears in the cooler distal areas, creating the distinctive pattern (Figure 5f–h) [5,35,36,37]. Although newborn colourpoint kittens appear almost entirely white, experienced breeders are often able to distinguish those with eumelanin-based (black/seal or blue) pigment from those with pheomelanin-based (red or cream) pigment, as the former tend to display slightly darker shading shortly after birth. Importantly, in colourpoint cats that produce black pigment, the colour is not described as black but is referred to as seal [1,2,3,8,9,10,11,12,13]. Under the interaction of other loci (e.g., *A*, *I* and *O*), the point may be of various colours or intensity (Figure 1c,d, Figure 3c,d, Figure 4d and Figure 5a–d).

An integral feature of the colourpoint phenotype is the blue eye colour, which is consistently associated with the *c^s^* allele. This trait arises from the same temperature-sensitive mechanism: because pigment formation is reduced in the iris, normal melanin deposition is absent, and light scattering within the unpigmented stroma produces the characteristic blue hue. The presence of blue eyes is therefore a direct consequence of incomplete pigment synthesis caused by the thermolabile tyrosinase and is a defining and desirable aspect of the colourpoint appearance [5,35,36,37,38].

At the molecular level, the *TYR* gene encodes tyrosinase, a copper-dependent oxidase that catalyzes the conversion of tyrosine to L-DOPA and its subsequent oxidation to dopaquinone, the initial steps of melanin biosynthesis. The *c^s^* mutation involves a single nucleotide substitution leading to an amino acid change that destabilizes the enzyme at temperatures above approximately 37 °C. Consequently, melanin production is spatially restricted to areas of lower temperature.

In addition to *C* (full colour) and *c^s^* (colourpoint), in different breeds, several other *TYR* alleles have been identified, such as *c^b^* (Burmese), *c* (complete albino, *tyrosinase-negative*), and *c*^2^ (albino, blue eyes with a reddish reflection), or newly discovered *c^m^* (mocha). Specific allelic variants differ in the degree of coat lightening on the body and the distinctiveness of points, as well as the lack of pigment. In the case of *c^s^*, the coat is not white, but depending on the base colour, it takes on colours ranging from light grey to beige, with the intensely coloured points contrasting with the lighter coat colour on the body. The phenotype determined by *c^b^c^b^* is characterized by less pigment reduction on the body, so the points do not stand out as much from the coat. An interesting aspect is the interaction of the *c^b^* and *c^s^* alleles, which in a heterozygous genotype give an intermediate phenotype. The mocha phenotype appears as a much lighter version of the Burmese, with less distinguished points and blue eyes [36,37,39,40]. However, only the *C* and *c^s^* alleles are recognized within the Siberian breed.

Within the Siberian cat, the *c^s^* allele has been present since the earliest development of the breed. Individuals expressing the colourpoint phenotype are traditionally referred to as Neva Masquerade, a name that reflects both the River Neva in St. Petersburg and the “masked” appearance formed by the darker facial points [5,41,42,43]. Genetically, Siberian and Neva Masquerade cats share the same gene pool and differ only in the expression of the *c^s^* allele. Feline organizations recognize the colourpoint variety as part of the Siberian breed standard [1,2,10,11,13]. However, the FIFe, the ACF, and the CCCA classify the Neva Masquerade as a separate but sister breed to the Siberian cat, while the remaining organizations do not distinguish between the two and regard them as a single breed [3,8,9]. The NZCF is the only organization that does not recognize the colourpoint variety as either part of the Siberian breed or as a sister breed Neva Masquerade [12]. Despite the differences in classification, interbreeding between traditionally coloured Siberians and colourpoint individuals is allowed and maintains the shared genetic foundation of these closely related populations.

#### 2.2.4. Locus D

**Figure 6:**
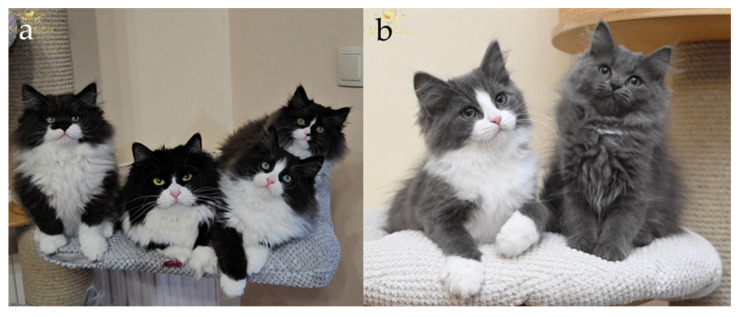
Dilution in Siberian cats, (**a**) black with white kittens, (**b**) blue and white kittens. The colour of the nose leather in blue cats is also dark, but lighter than in black cats and harmonizes with the colour of the fur. Source of photographs: cattery Blue Chelsea.

Dilution (Locus *D*) is a recessive trait that lightens the visual effect of eumelanin (black/brown) and pheomelanin (red/yellow). In practice, this means that black coat turns into blue (grey) (Figure 6a,b) and red appears as cream (Figure 1a,b), while the underlying solid or tabby pattern remains intact [27,44,45]. Generally, in domestic cats, dilution can also convert chocolate to lilac and cinnamon to fawn; however, these colours (chocolate, lilac, cinnamon, fawn) are not explicitly recognized in the Siberian breed by major registries. Only in the TICA organization chocolate and cinnamon colours, as well as their dilutions, are not prohibited in the breed [2]. Therefore, when discussing Locus *D* in Siberians, we are mainly describing the black-to-blue and red-to-cream transformations.

At the molecular level, feline dilution results from loss of function in melanophilin (*MLPH*), the gene at Locus *D* [27,44]. *MLPH* encodes a cargo linker component of the RAB27A–MLPH–myosin VA (MYO5A) melanosome transport complex, which moves pigment granules along actin filaments from the melanocyte cell body to the tips of its dendrites [27,44,45]. When MLPH is defective, melanosomes tend to cluster around the nucleus instead of being evenly distributed, causing pigment deposition to form fewer, larger, and irregularly spaced aggregates as the hair shaft grows, giving the coat a visibly “diluted” appearance [27,44,45].

Linkage and association mapping identified the feline dilution locus on chromosome C1 and pinpointed a frameshift single-base deletion in exon 2 of *MLPH* (c.83delT; p.L28Rfs*12) as the cause of the standard blue/cream dilution [46]. This allele is fully penetrant and autosomal recessive: cats with *D*/*D* or *D*/*d* show full-intensity colour, while *d*/*d* cats are dilute (blue or cream in Siberians) [27,44]. Further fine-mapping and exome-based resources confirmed this variant and updated its genome coordinates on current assemblies (e.g., recorded in trait panels based on the *Felis catus* reference genome) [46].

Histological studies support this model of melanosome transport. Prieur and Collier [45] examined hair shafts from various coat-colour dilution phenotypes, including blue, smoke, and pink-eyed dilution cats. Black hairs contained many small, dark melanin granules evenly spread along the shaft, while blue hairs (dilute) showed larger melanin granules, some very big but fairly regularly shaped, with uneven spacing [45]. Conversely, “smoke” hairs (a dominant inhibitor phenotype unrelated to Locus *D*) had few melanin granules in the basal part of the hair but otherwise normal size and distribution [45]. Genomic and population studies show that the *MLPH*:c.83delT dilution allele is common in the domestic cat gene pool. Large-scale genotyping has revealed that this variant is present in Siberian cats, as well as in many other breeds and random-bred populations, indicating an ancient origin that spread through domestication and breed development [6,46,47,48,49].

#### 2.2.5. Locus I

**Figure 7:**
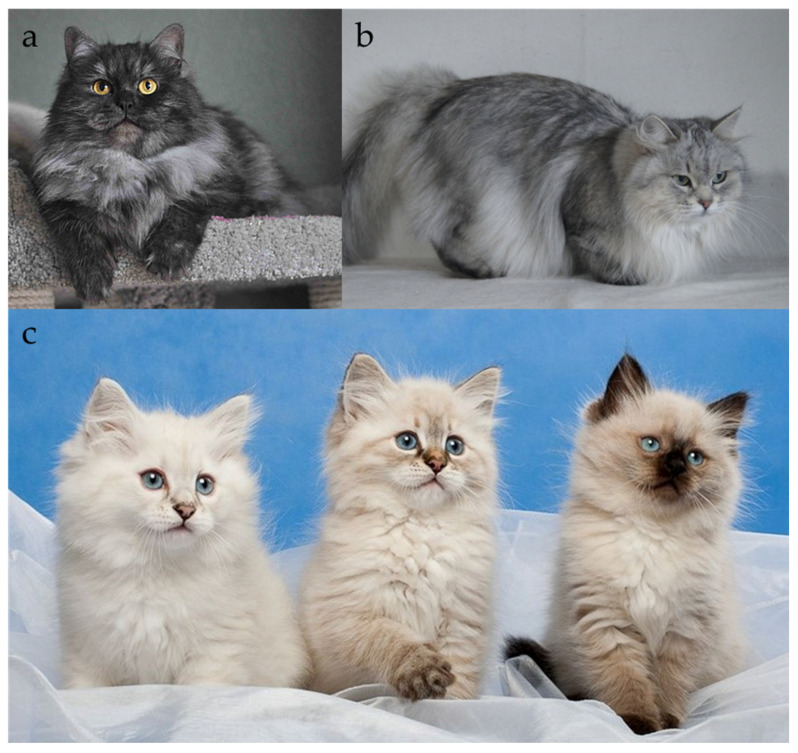
Silver and smoke Siberian cats, (**a**) black smoke, (**b**) black silver tabby, (**c**) from the right—seal point, seal tabby point, seal silver tabby point. Source of photographs: (**a**) cattery Blue Chelsea, (**b**) cattery od Ivanki, (**c**) cattery Błękitny Anioł.

The silver coat phenotype in domestic cats, traditionally referred to as the “Inhibitor” or “silver“ locus (*I*), is characterized by a marked reduction in pigment deposition along the hair shaft. The basal portion of the hair is depigmented and appears white or near-white, while pigmentation is retained only at the distal tip. This results in the distinctive silvery or shaded appearance of the coat, often described as “tipped” or “chinchilla,” depending on the proportion of the pigmented region. In silver tabby cats, the ground colour is pale silver-grey, contrasting with darker tabby markings and a bright undercoat (Figure 7b). In red and cream cats, the same mechanism produces warm, pale coats with slightly reddish tips, commonly referred to as “cameo.” When the “Inhibitor” allele is expressed in non-agouti cats (*a*/*a*), the coat displays a dark surface with a pale undercoat visible when the fur is parted; this variant is known as “smoke” (Figure 7a) [1,2,3,7,8,9,10,11,12,13,50].

The inheritance of the silver phenotype is controlled by a single autosomal dominant locus, designated as “Inhibitor” (*I*). Early breeding reports suggested that homozygous individuals (*I*/*I*) might exhibit a more intense or extensive silver expression compared to heterozygotes (*I*/*i*); however, this hypothesis has not been substantiated by genetic or phenotypic data. The “Inhibitor“ allele acts as a pigment distribution modifier rather than altering melanin synthesis directly, and affects both eumelanin and pheomelanin, although the suppression of eumelanin is typically more visually pronounced [7,50].

Within the spectrum of silver-based phenotypes, three principal gradations are recognized: silver, shaded, and shell (chinchilla), which differ primarily in the proportion of the hair shaft occupied by pigment [1,2,3,8,9,10,11,12,13]. In the “silver” phenotype (often referred to as “silver tabby”), approximately one-half of each agouti hair is pigmented, producing a clearly defined tabby pattern against a pale silver background. In “shaded silver” cats, the pigment occupies roughly one-third of the hair length, resulting in a softer, shaded appearance in which the tabby pattern is partially obscured but still perceptible, especially on the head, legs, and tail. The most extreme form, “shell” or “chinchilla”, is characterized by pigment restricted to only the distal one-eighth to one-tenth of each hair, yielding a nearly white coat with a subtle veil of colour visible on the back, flanks, and tail [1,2,3,8,9,10,11,12,13].

Traditionally, these gradations have been attributed to the modifying action of the hypothetical “wide band” (*Wb*) locus, which was proposed to regulate the width of the depigmented (pale) zone in agouti hairs. However, the existence and molecular identity of the *wide band* gene remain unconfirmed, and its precise mode of inheritance continues to be debated. Interestingly, in Siberian cats, the co-occurrence of the Inhibitor (silver) allele and the recessive “wide band” variants associated with the “sunshine” phenotype has been shown to produce a mixed coloration known as “silver sunshine”, in which golden and silver hair segments coexist within the same coat. This phenomenon contradicts the classical wide-band hypothesis, which assumes mutual exclusivity between the genetic mechanisms underlying silver and golden pigmentation.

Menotti-Raymond et al. [51] conducted a genome-wide linkage analysis in pedigreed silver and non-silver cats and mapped the Inhibitor locus to a specific region on feline chromosome D2, between the markers FCA678 and FCA700, spanning approximately 3.5 cM. This interval does not overlap with known pigmentation genes such as *TYR*, *TYRP1*, *MC1R*, Receptor Tyrosine Kinase (*KIT*), or Premelanosome Protein (*PMEL*), indicating that the silver effect is likely governed by a novel gene influencing the spatial or temporal regulation of pigment deposition along the hair shaft. The authors proposed that the gene responsible for the “Inhibitor” effect may modulate melanosome transport, pigment transfer to keratinocytes, or the timing of melanin synthesis during hair growth. Interestingly, a similar hypopigmentation phenotype is observed in mice carrying the “pearl” (*pe*) mutation in the Adaptor Related Protein Complex 3 Subunit Beta 1 (*Ap3b1*) gene. Although the visible effect—a lightened hair coat—is reminiscent of the silver phenotype in cats, the underlying genetics differ: “pearl” is inherited as a recessive trait and primarily affects melanosome trafficking within cells, whereas the feline “Inhibitor” allele is dominant [52]. Thus, while the mouse “pear”l coat resembles feline silver in appearance, the molecular mechanisms and inheritance patterns are distinct.

Laboratories in the USA and China have attempted to identify the genetic basis of the silver phenotype in cats [53]. In May 2023, the Chinese laboratory Petgeno reported the identification of mutations associated with silver and developed a test that was initially limited to China but became accessible to European breeders in August 2023 [54]. Concurrently, research at the University of Missouri aimed to establish a reliable assay for the silver trait; however, a subset of phenotypically silver cats did not carry the identified mutations [55]. Both groups have yet to publish peer-reviewed results, and the University of Missouri test is not yet incorporated into standard commercial testing panels due to technological limitations. These ongoing efforts suggest that, although causal mutations for some silver cats have been proposed, additional allelic variants, potentially recessive, may contribute to the full spectrum of the silver phenotype. All cat organizations recognize the silver colour in the breed [1,2,3,8,9,10,11,12,13].

#### 2.2.6. Locus Wb

**Figure 8:**
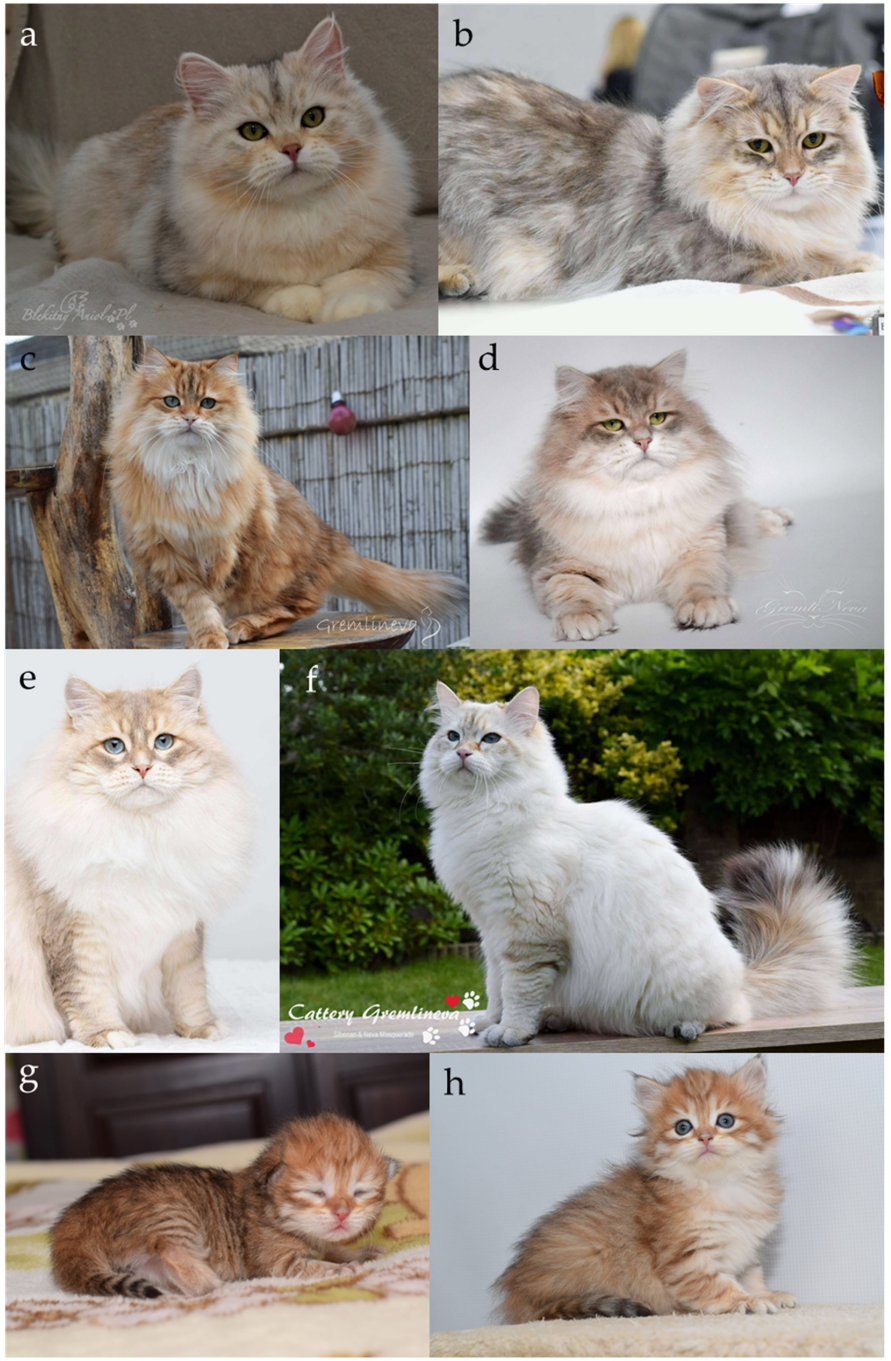
Sunshine Siberian cats. (**a**) Black silver sunshine tabby (bimetallic), (**b**) blue silver sunshine tabby (bimetallic), (**c**) black sunshine tabby, (**d**) blue sunshine tabby, (**e**) blue sunshine tabby point, (**f**) seal sunshine tabby point, (**g**,**h**) black sunshine tabby, the same kitten, but at a different ages. Sunshine cats have pink nose devoid of the dark outline typically seen in standard tabby cats. Source of photographs: (**a**) cattery Bękitny Anioł, (**b**–**h**) cattery Gremlineva.

The Siberian cat breed exhibits a distinctive coat colour phenotype, referred to as *sunshine*, which has attracted considerable attention due to its characteristic golden hue and atypical inheritance pattern (Figure 8a–h). These cats show a warm-toned undercoat, a reduction in the intensity of the tabby markings, an expanded cream-to-white region surrounding the nose that often extends onto the chest, and a pink nose devoid of the dark outline typically seen in standard tabby cats. The *sunshine* phenotype occurs exclusively in agouti cats (agouti modifier). Although certain feline registries recognize the “golden” coloration in this breed, molecular and phenotypic evidence suggest that its genetic basis differs from that described in Persians and British Shorthairs [56].

Historically, the golden phenotype was first documented in Persian cats, where it was attributed to the “wide-band” (*Wb*) effect. In Persians, the golden colour is thought to be caused by a recessive (or dominant) “wide-band” allele (*wb*) that acts as a modifier of the agouti pattern [7]. The *Wb* allele broadens the pheomelanin-rich (pale brown) band at the base of each agouti hair while restricting eumelanin deposition to the terminal region of the shaft, thereby producing the characteristic warm golden tone. The paw pads in such cats are typically pink or correspond to the colour of the hair tips. It is generally assumed that golden Persians carry the *Wb* allele and either lack the silver inhibitor gene (*I*) or possess a recessive form of it. Moreover, the “chinchilla” and “tipped” (shell) phenotypes in Persians are believed to result from the interaction of silver coloration with homozygosity for the “wide-band” allele [51,57]. This premise implies that a cat cannot simultaneously express both golden and silver coat coloration.

**Figure 9:**
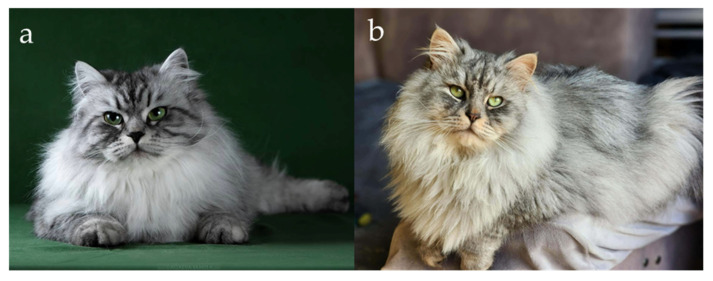
Pictures of two black silver tabby cats: (**a**) black silver tabby without rufism (“pure” silver in cold tone), (**b**) black silver tabby with rufism (warm tone, visible yellowish colour in nose, chin, cheeks). Source of photographs: cattery Matryoshka.

Breeders of Siberian cats have reported the occurrence of individuals exhibiting an unusual coat pattern characterized by the coexistence of both silver and golden regions within the same pelage. Initially, such cats were believed to exhibit an extreme degree of rufism, a trait that determines the intensity of reddish or brownish pigmentation in agouti-patterned coats [7,57,58]. Rufism accounts for the continuous variation ranging from warm brown to cool grey tones in tabbies (non-agouti cats do not exhibit traits of rufism), and from deep red to pale orange in red cats. The wide range of phenotypic expression associated with rufism suggests that this trait may be polygenic in nature, potentially arising from the combined effects of multiple genetic factors. In silver cats displaying rufism, this trait is considered a colour fault by many breed associations, as a pure cool silver tone is preferred, the difference between silver roufistic and non-rufistick silver cat is clearly visible on Figure 9a,b. However, in Siberians, the extensive golden regions observed among silver hairs could not be adequately explained by rufism alone. Cats expressing this phenotype were informally termed “bimetallic” owing to their distinctive two-tone appearance (Figure 8a,b) [59].

Subsequent breeding observations revealed that certain silver Siberians developed small, golden-brown patches, which reappeared in their offspring, even when both parents exhibited a silver phenotype. Although somatic chimerism was initially hypothesized, the consistent recurrence of this trait across generations supported a recessive mode of inheritance distinct from the Persian “wide-band” system [59]. Females displaying the bimetallic pattern were occasionally misclassified as tortoiseshells; however, the absence of the red (*O*) allele and the presence of dark pink nasal leather indicated that they were not true genetic tortoiseshells.

The term “golden,” historically used by Siberian breeders, conflicted with the nomenclature applied to the “wide-band golden” phenotype in Persians and British Shorthairs. Consequently, the designation “sunshine” was adopted to describe this distinct form of golden coloration [56,59]. The sunshine phenotype bears a superficial resemblance to the “amber” coloration in Norwegian Forest Cats and the “carnelian” (also known as “serdolic” or “copal”) variant in Kurilian Bobtails, both caused by mutations in the melanocortin 1 receptor gene [14,60]. However, molecular analyses have excluded *MC1R* mutations in sunshine-coloured Siberians. At that time, genetic tests for the wide-band and silver inhibitor loci were unavailable, and identification of the sunshine phenotype relied solely on phenotypic evaluation and pedigree analysis.

In 2021, a genome-wide association study combined with homozygosity mapping identified a single genomic region associated with the sunshine phenotype in Siberian cats [56]. Within this region, the Corin, serine peptidase (*CORIN*) gene was identified as a strong candidate, as *CORIN* variants had previously been linked to golden phenotypes in mice and tigers, and the gene acts as a known modifier of the *ASIP* pathway [61,62]. A homozygous missense variant, *CORIN:c.2383C>T*, resulting in the amino acid substitution p.(Arg795Cys), was detected in sunshine Siberians. The segregation pattern of this variant was consistent with autosomal recessive inheritance, and the allele was not detected in 13 other cat breeds, except for isolated carriers in Kurilian Bobtails and ToyBobs. Microscopic analysis of the hair structure in affected Siberians revealed an elongated pheomelanin band consistent with prolonged *ASIP* activity, supporting the hypothesis that this variant represents the Siberian recessive wide-band allele (*wbˢⁱᵇ*) [56].

A subsequent study, published in 2022, identified a second missense variant in the same gene, *CORIN:c.839G>A*, in Siberians displaying an intensified golden tone, referred to as the “extreme-sunshine” phenotype [63]. This variant was proposed to represent the Siberian recessive extreme wide-band allele (*wbᵉˢⁱᵇ*). The resulting dominance hierarchy among the “wide-band” alleles was established as: *Wb^+^* > *wbᵉˢⁱᵇ* > *wbˢⁱᵇ*. These findings provided molecular evidence that the sunshine phenotype in Siberian cats arises from breed-specific *CORIN* variants, distinguishing it genetically and phenotypically from the classic “wide-band golden” found in Persians. In 2022, the variant of CORIN gene was also identified as responsible for the golden coat coloration in British Shorthair cats [64].

The “sunshine” coat modification was formally recognized by the World Cat Federation (WCF) in 2017, assigned under the coat colour code “u,” and restricted exclusively to Siberian cats [1]. In 2025, the “sunshine” colouration was provisionally accepted by the Fédération Internationale Féline (FIFe), limited to individuals expressing eumelanin (black-based) pigmentation, with the condition that the presence of the *CORIN* variant must be confirmed by molecular genetic testing [65,66,67]. Cats exhibiting the “bimetallic” coat coloration are assigned the coat color code “us.” The “golden” coloration is recognized by all major international feline federations with the exception of the New Zealand Cat Fancy (NZCF) and the Southern African Cat Council (SACC). Within the Governing Council of the Cat Fancy (GCCF), the golden phenotype is registered under the term “Zolotoy”, while the Australian Cat Federation (ACF) and the Cat Fanciers’ Association (CFA) also recognize the “bimetallic” colour variant [1,2,3,8,9,10,11,12,13].

#### 2.2.7. Locus W (S)

For years, breeders distinguished between two loci related to white coloration: *S* and *W*. It is now known that both white spotting (formerly known as locus *S*) and dominant white (*W*) are associated with the same gene of Receptor Tyrosine Kinase (*KIT*) and will therefore be discussed as locus W.

**Figure 10:**
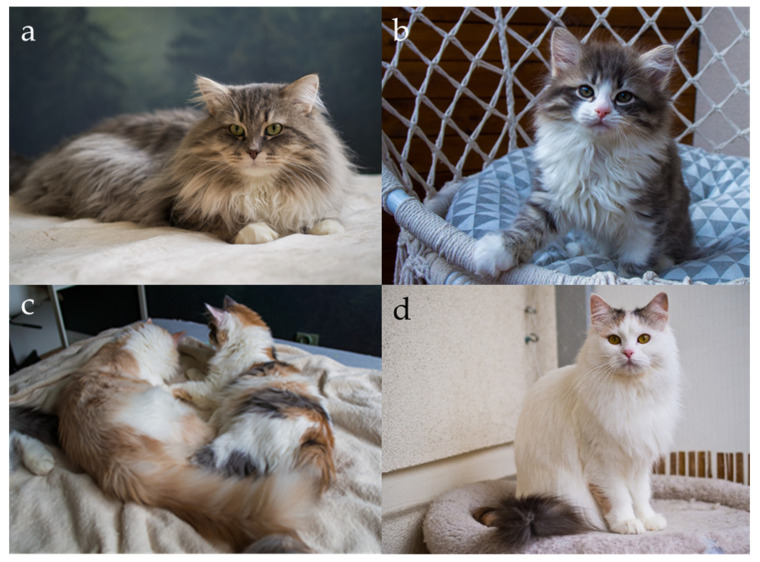
White patching Siberian cats, (**a**) up to 25% of white (white spotting limited to the gloves), (**b**) 25–50% white (on the legs, face and belly), (**c**) 50–75% (colour patches on the heads, backs and tails), (**d**) more than 75% (colour limited to 2 patches on the head, one patch on the front leg and tail). The presence of a white patch surrounding the nose results in an overall pink coloration of the nasal leather. Source of photographs: cattery First Snow.

White spotting (*W^S^*, often referred as locus S by breeders) is a well known pigmentation pattern in the domestic cat, characterized by the presence of unpigmented, white areas of fur that vary in size and distribution across the body (Figure 1a,b,f, Figure 6a,b and Figure 10a–d) [7]. These patches result from an incomplete migration of melanocyte precursors (melanoblasts) from the neural crest during embryonic development, leading to regions of skin and hair follicles devoid of melanin-producing cells [68]. Phenotypically, white spotting ranges from minimal white markings, such as a small spot on the chest or paws, to nearly complete depigmentation, where only small, coloured areas remain (Figure 10a–d). The degree of expression is often classified along a gradient from low white to high white, with the most extensive forms approaching a fully white phenotype, though genetically distinct from cats carrying the dominant white allele, which breeders often refer to as locus *W* [7]. 

Breeders and early geneticists noted that the presence and extent of white markings followed a heritable pattern, leading to the postulation of a dominant allele *W^S^* (with variable expressivity and incomplete penetrance. Robinson and colleagues [7] formalized this concept within the classical framework of feline coat colour genetics in the mid-twentieth century. The segregation of alleles became evident in certain breeds, such as Birman, Turkish Van, Turkish Angora, Ragdoll, and Snowshoe cats, where white spotting was selectively maintained to produce characteristic phenotypic patterns [7,69]. With the rise in molecular genetics at the turn of the twenty-first century, the *W^s^* locus was mapped to the *KIT* gene, a finding that parallel discoveries in other mammalian species such as horses, mice, and dogs, where *KIT* mutations similarly produce piebald or white-spotting phenotypes [70,71,72,73].

At the molecular level, the white spotting pattern in cats results from alterations that affect the expression or function of the *KIT* gene, which encodes a receptor tyrosine kinase essential for the proliferation, survival, and migration of melanoblasts during embryogenesis. In cats exhibiting white spotting, an insertion of a retrotransposon within intron 1 of *KIT* has been identified as the causal variant, disrupting normal transcriptional regulation and leading to a patchy absence of melanocytes in the skin [74,75,76]. For decades, it was assumed that homozygous *W^S^*/*W^S^* (previously *S*/*S*) individuals display a higher proportion of white areas compared to heterozygotes *W^S^*/*w* (previously *S*/*s*). However, this relationship has not been empirically confirmed, and pedigree analyses have suggested that an additional, as yet unidentified factor must influence the expression of white spotting in domestic cats [77]. Furthermore, molecular studies have shown that some Siberian cats also carry the *w^g^* allele, which is responsible for the distinctive white glove characteristic of Birman cats. This phenotypic effect is attributed to the presence of an indel mutation (c.1035_1036delinsCA) in *KIT* [6,28,69]. However, breeders and breeding associations do not distinguish between the *W^S^* and *w^g^* alleles, and cats with both white spotting and gloves are referred to as “with white”.

A further practical complication arises from the inconsistent terminology used by various feline organizations to describe phenotypes associated with different degrees of white spotting. Major cat federations apply divergent percentage thresholds for classifying bicolour, harlequin, and van patterns, which can lead to inconsistencies in registration and pedigree documentation, particularly when cats are transferred between organizations. For example, within the Fédération Internationale Féline (FIFe), harlequin is 50–75% white on the body, while the “van” pattern is precisely described as “two colour patches in the face separated by a white blaze, one patch commences on the rump and ends on the tip of the tail. No white hairs on the solid colour parts. Three small irregularly distributed colour patches on the body and/or on the legs are to be tolerated” [3]. This precise morphological definition means that cats with more than 75% white coat but without the specific patch distribution required for “van” classification may not fit any category unambiguously. This situation often creates confusion among breeders when determining colour codes, especially in cats showing extensive white without meeting the strict pattern criteria. In contrast, the World Cat Federation (WCF) defines “bicolour” as “1/3 till 1/2 white”, “harlequin” as “1/6 colour and 5/6 white,” and “van” as “1/8 colour only on the head and tail, and 7/8 white” [1]. Additional discrepancies are evident in American federations. The International Cat Association (TICA) describes a “locket” as “a small distinct spot of white usually found on the chest, abdomen (belly), groin, or armpit areas, believed to be the result of a white spotting gene that is different from the dominant white spotting gene,” and cats with a locket are formally classified as “without white” [2]. Similarly, the Cat Fanciers’ Association (CFA) specifies that “cats with buttons, spots and/or lockets shall be judged as their basic colour with no penalty for such locket, spot and/or button [10]. However, there is no empirical confirmation that the occurrence of lockets is caused by a separate gene from the known *W^S^* allele, and current genetic evidence does not support the existence of a distinct “locket gene.” Terminological differences extend further into how federations categorize and name colours involving white. In FIFe, WCF, and ACF, the officially recognized categories include “unspecified amount of white” (“with white”), “bicolor”, “harlequin”, and “van” [1,2,8]. In TICA and CFA, color names are supplemented by the terms “with white” or “and white”, while also distinguishing between “bicolour” and “van” patterns as separate variants [2,10]. Similarly, the CCCA and NZCF systems add “and white” to the color description [9,12]. In the GCCF, the terminology includes “with white”, “bicolour”, and “high white”, whereas the SACC does not provide explicit information regarding the classification of cats with white spotting [11,13]. The coexistence of these inconsistent systems complicates phenotype-based registration and may contribute to discrepancies in pedigree databases across federations.

**Figure 11:**
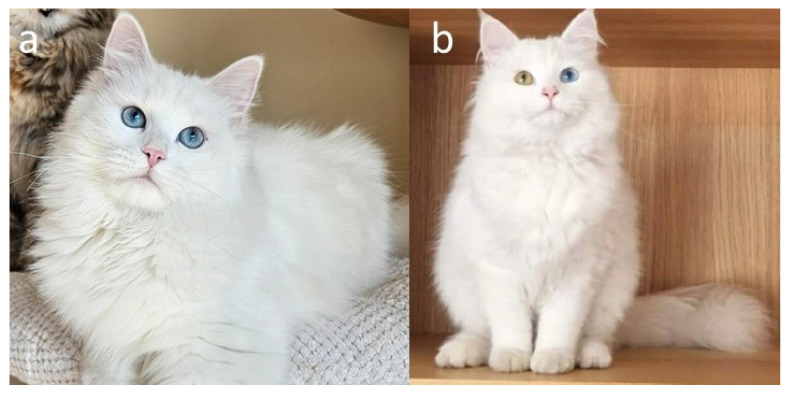
White Siberian cats: (**a**) cat with blue eyes—pedigree analysis revealed that this individual carries the *colourpoint* genotype masked by the dominant white coat, which explains the presence of blue eye colour; (**b**) odd-eyed cat. Source of photograph: (**a**) cattery Blue Chelsea, (**b**) cattery de Nice.

The dominant white (*W*) phenotype in domestic cats is characterized by complete depigmentation of the hair and skin, resulting in an entirely white coat (Figure 11a,b). Unlike the white spotting (*W^S^*) allele, which causes localized absence of pigmentation, the *W* allele completely inhibits the migration and survival of melanocyte precursors (melanoblasts) during embryogenesis [7,68]. As a consequence, the hair follicles and skin lack melanin-producing cells throughout the body. Despite the external appearance of uniform whiteness, *W*/- cats possess an underlying genetic colour and pattern that remain masked by the dominant white gene. If not for this allele, such cats would exhibit the full expression of their genetically determined coat colour and pattern. White cats carrying the *W* allele may have blue, copper, gold, green, or odd-coloured eyes. The variation results from the suppression or uneven distribution of melanocytes in the iris, with blue eyes indicating a complete absence of pigment, while copper or gold eyes reflect residual melanin production.

A distinctive feature often observed in kittens carrying the *W* allele is the presence of small, dark-coloured spots or smudges on the top of the head. These transient markings typically reflect residual areas of melanocyte activity that disappear as the cat matures. The phenomenon is thought to result from incomplete inhibition of melanoblast migration during early development. Such spots usually vanish after the first moult, leaving a completely white adult coat.

From a breeding perspective, the production of dominant white cats carries notable health risks. The *W* allele is pleiotropic and not limited to pigmentation pathways; it also affects structures derived from the neural crest, including components of the inner ear. Consequently, a significant proportion of white cats with blue eyes suffer from congenital sensorineural deafness (CSD). This condition, analogous to Waardenburg syndrome in humans, arises from the absence or degeneration of melanocytes in the stria vascularis of the cochlea, leading to impaired endolymph homeostasis and permanent hearing loss. The prevalence of deafness varies depending on eye colour and genetic background, white cats with two blue eyes are the most severely affected, while those with one blue and one non-blue (odd-eyed) show unilateral or partial hearing loss less frequently [78,79,80,81,82,83]. Breeding two white individuals is therefore discouraged, as it increases the risk of producing deaf offspring and may also impact litter viability. Responsible breeding programmes typically include mandatory auditory testing (BAER—Brainstem Auditory Evoked Response) before using white cats for reproduction.

In contrast, the relationship between white spotting in the absence of dominant white and the risk of CSD remains less clearly defined in the peer-reviewed feline literature. Although white spotting involves the *KIT* gene and is mechanistically compatible with melanocyte-related inner ear effects, strong population-level data directly linking white spotting alone to an increased deafness risk in cats are limited. Therefore, dominant white continues to be the most consistently documented pigmentation-associated risk factor for CSD [84,85]. Importantly, to date, no peer-reviewed studies have specifically evaluated the prevalence of congenital sensorineural deafness in Siberian cats, whether related to dominant white or graded white spotting phenotypes.

At the molecular level, the *W* locus corresponds to mutations in the *KIT* gene, located on feline chromosome B1 [74,75,76]. *KIT* encodes a transmembrane receptor tyrosine kinase that binds stem cell factor (SCF) and plays essential roles in melanoblast proliferation, survival, and migration, as well as in hematopoiesis and gametogenesis. The dominant white phenotype in cats is caused by a structural variant, a large insertion of a feline endogenous retrovirus type 1 (*FERV1*) within intron 1 of *KIT*. This insertion disrupts normal gene regulation and transcription, resulting in the total absence of functional melanocytes throughout the skin and coat. Interestingly, the same gene is implicated in the piebald (*W^S^*) phenotype, but different mutations or regulatory mechanisms lead to partial rather than complete depigmentation. Thus, *KIT* represents a classic example of a pleiotropic gene in which distinct mutations produce a continuum of pigmentary phenotypes, from mild spotting to complete whiteness.

Cat registries recognize the occurrence of white coat colour in Siberian cats. Some federations, including the World Cat Federation (WCF), Fédération Internationale Féline (FIFe), and the Governing Council of the Cat Fancy (GCCF), include in their breeding requirements the stipulation that white breeding cats must possess a veterinary certificate confirming normal hearing before being used for reproduction [1,3,11].

### 2.3. Eye Pigmentation

**Figure 12:**
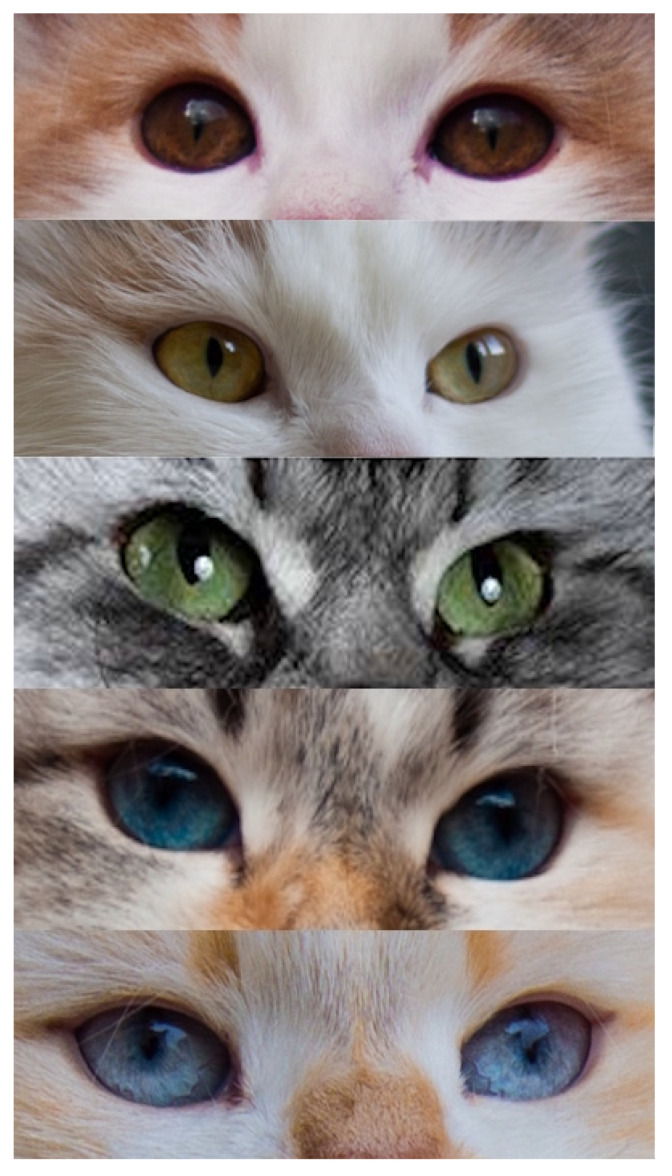
Colours of Siberian cats eyes, from the top: dark orange, yellow, green eyes in Siberian cats, dark blue and light blue in Neva Masquerade (colourpoint Siberians). It should also be noted that all kittens are born with blue eyes, as melanin deposition in the iris is not yet complete at birth. Eye colour gradually changes during postnatal development as pigmentation increases, and the final eye colour may not be fully established or reliably assessed until late juvenile or even adult stages. Source of photograph: catteries First Snow and Matryoshka (green eyes).

Eye colour in domestic cats reflects the amount and distribution of melanin within the iris and associated ocular structures, with lighter eye colours resulting from reduced melanin deposition and increased optical scattering. In most non-colourpoint Siberian cats, eye colour is typically described in breed standards and by registries using categories such as green and orange (often referred to as yellow or gold), with the latter terminology predominating in the standards of many feline organizations (Figure 12). This variation is generally considered a complex, polygenic trait, and no single major locus equivalent to those controlling coat colour has been identified as a reliable predictor of eye colour in Siberian cats. Consequently, eye colour is not typically used as a primary criterion in routine breeding decisions beyond compliance with breed standards [86].

In contrast, the colourpoint phenotype (*c^s^*/*c^s^*), caused by temperature-sensitive tyrosinase activity, is consistently and obligatorily linked to blue eyes. This link stems from reduced ocular pigmentation caused by impaired tyrosinase-dependent melanogenesis, i.e., lower melanin in the iris increases light scattering, giving the characteristic blue appearance. The shade of blue can vary, from very pale blue to deeper blue (Figure 12). Darker blue eyes are generally preferred by breed standards, and some breeding programmes actively select for more intense blue shades. Empirical breeding experience shows that targeted pairings can raise the chances of producing offspring with darker blue eyes, supporting the idea that eye colour intensity is a heritable, quantitatively variable trait rather than a fixed result of the cs allele alone [87].

Notably, red (pheomelanin-based) colourpoint cats consistently exhibit paler blue eye colour than eumelanin-based colourpoints. This observation is consistent with the broader role of melanin type in ocular pigmentation, as pheomelanin contributes less effectively to ocular pigment density than eumelanin. Thus, even within the *c^s^*/*c^s^* genotype, the base coat colour influences the final eye colour phenotype. In Siberian cats, individuals that appear phenotypically white but exhibit blue eyes most commonly represent colourpoint cats (*c^s^*/*c^s^*) whose coat colour is masked by the dominant white allele (*KIT*/*W*). In such cases, blue eye colour reflects the underlying colourpoint genotype rather than albinism or a separate eye-colour determinant. This distinction is of practical importance in pedigree analysis and breeding documentation [88].

**Figure 13:**
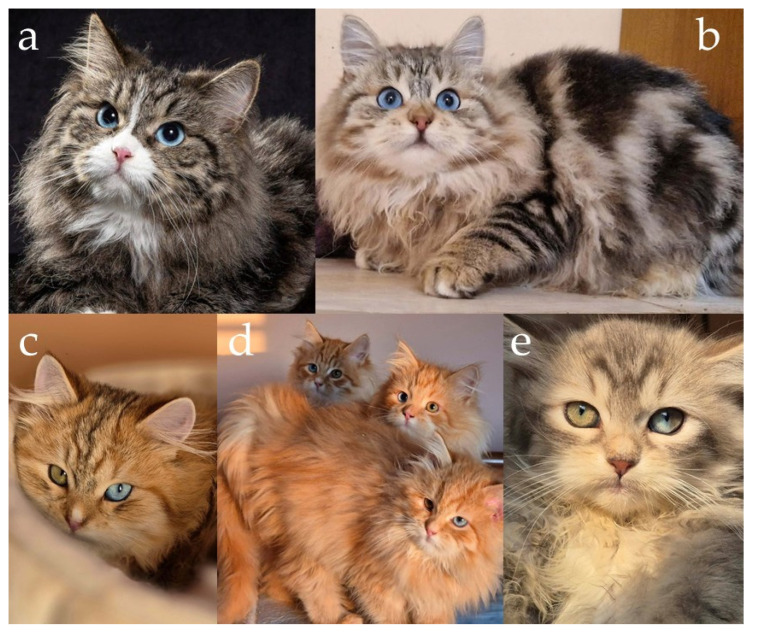
Dominant blue eyes, (**a**) both blue eyes, (**b**) both blue eyes (visible minimal white on the toe), (**c**) odd eyes, (**d**) three kittens with odd eyes, (**e**) particolored eyes. Source of photograph: (**a**) Great Joy cattery, (**b**–**e**) Coccole and Fusa cattery.

Cats with extensive depigmentation caused by *KIT* (dominant white) may show blue, orange/gold, green, copper, or odd-eyed phenotypes. The odd-eyed look is generally seen as a developmental mosaic, where uneven distribution of melanocytes between the left and right irises causes one depigmented (blue) eye and one pigmented eye. Although odd-eyed or blue-eyed phenotypes are most often associated with extensive white or colourpoint genotypes, recent molecular studies have demonstrated that blue eyes can also occur in cats with minimal or no visible white spotting and without TYR-dependent colourpoint patterning (Figure 13) [89]. This phenotype, commonly referred to as dominant blue eyes (DBE), is characterized by one or two blue eyes or sectorial heterochromia and is often accompanied by little white spotting. Molecular analyses have demonstrated that DBE is a genetically distinct form of ocular hypopigmentation that is independent of both *KIT* and *TYR* pathways [89].

Genetically, DBE is heterogeneous and has been associated with multiple independent pathogenic variants affecting the Paired Box 3 (*PAX3*) gene, which is a key regulator of neural crest development and melanocyte migration. To date, multiple distinct DBE-associated alleles have been identified in various feline lineages, including intronic retroviral insertions and other structural or regulatory variants that influence *PAX3* expression. This suggests multiple independent origins rather than a single shared mutation [89,90,91].

Under low-light conditions, eye shine observed in cats results from the tapetum lucidum, a reflective layer located behind the retina. The apparent colour of this reflection (usually greenish in many cats) depends on eye pigmentation and retinal structure and should not be mistaken for iris colour itself. In colourpoint cats, variations in eye pigmentation may change the perceived color of the reflected light; however, this effect is due to retinal optics rather than iris pigmentation alone.

## 3. Conclusions

A significant part of the knowledge on the genetic determinants of colour variations in Siberian cats is based on earlier studies of other breeds. However, dissimilarities can be observed between different breeds, for example, the absence of specific varieties in the breed (amber or russet), as well as the occurrence of colours characteristic mainly of Siberian cats, like sunshine. Thanks to these differences, in recent years the breed has become the subject of interesting research that has helped to explain the molecular basis of selected coat colours (Table S1). Yet some of them still remain insufficiently resolved at the molecular level. Continued genomic research, including whole-genome association studies and functional analyses, will be essential for clarifying these mechanisms.

Another aspect is comparative assessment of major feline registries, which revealed persistent inconsistencies in nomenclature, acceptance of colour varieties, and classification rules (Table S2). Additionally, the dual use of the English term white spotting to describe white-spotting phenotypes, and the term spotted to denote a pattern type, contributes to significant ambiguity; this issue could be mitigated by adopting the term piebald for white spotting in standardized nomenclature systems. Such discrepancies influence breeding strategies, complicate international registration, and hinder transparent communication between organizations. Harmonization of terminology and the establishment of unified phenotype definitions, supported by genetic evidence, would significantly improve clarity in breeding practice and facilitate cross-registry collaboration.

Overall, the Siberian cat represents a genetically diverse breed in which colour phenotypes offer a valuable model for studying pigmentation genetics. Integrating molecular findings with standardized nomenclature frameworks will enhance both scientific understanding and practical breed management. Future efforts should focus on coordinated data sharing, expanded molecular testing, and the development of consensus guidelines that bridge the gap between genetic research and registry policy.

## Data Availability

No new data were created or analyzed in this study. Data sharing is not applicable to this article.

## Supplementary Materials

The following supporting information can be downloaded at https://www.mdpi.com/article/10.3390/genes17020208/s1. Table S1: Alleles recognized in the Siberian cat breed. Table S2: Nomenclature of coat colour varieties according to FIFe and WCF standards.

## Abbreviations

The following abbreviations are used in this manuscript:

ACF	Australian Cat Federation
ACTH	Adrenocorticotrophic hormone
Ap3b1	Adaptor Related Protein Complex 3 Subunit Beta 1
ARHGAP36	Rho GTPase Activating Protein 36
ASIP	Agouti Signalling Protein
BAER	Brainstem Auditory Evoked Response
cAMP	Cyclic adenosine monophosphate
CCCA	Co-Ordinating Cat Council of Australia
CFA	Cat Fanciers’ Association
CORIN	Corin, serine peptidase
CREB	CRE-binding protein
CSD	Congenital sensorineural deafness
DBE	Dominant blue eyes
DKK4	Dickkopf Wnt Signalling Pathway Inhibitor 4
Edn3	Endothelin 3
FERV1	Feline Endogenous Retrovirus Type 1
FIFe	Fédération Internationale Féline
GCCF	Governing Council of the Cat Fancy
KIT	Receptor Tyrosine Kinase
L-DOPA	L-3,4-dihydroxyphenylalanine
LVRN	Laeverin
MC1R	Melanocortin 1 Receptor
MITF	Microphthalmia-associated transcription factor
MLPH	Melanophilin
MYO5A	Myosin VA
NZCF	New Zealand Cat Fancy
PAX3	Paired Box 3
PCR	Polymerase chain reaction
PKA	Protein Kinase A
PMEL	Premelanosome Protein
RAB27A	RAB27A, Member RAS Oncogene Family
SACC	Southern African Cat Council
SCF	Stem Cell Factor
TAQPEP	Transmembrane Aminopeptidase Q
TICA	The International Cat Association
TYR	Tyrosinase
TYRP1	Tyrosinase-Related Protein 1
USSR	Union of Soviet Socialist Republics
WCF	World Cat Federation
WNT	Wingless/Int-1 (Wnt) signalling pathway
α-MSH	α-Melanocyte Stimulating Hormone
