# Supplementary material for: Colour Variants in Siberian Cats: A Comprehensive Review of Phenotype, Genetics, and Breed Registry Standards
Source: Genes (Basel). 2026 Feb 9;17(2):208. doi: 10.3390/genes17020208 (PMC12941195; doi:10.3390/genes17020208)
Supplement: Supplementary file 1 [file genes-17-00208-s001.zip › genes-4018633-supplementary.pdf]

Table S1. Alleles recognized in the Siberian cat breed.

Gene	Protein	Locus name used by breeders	Allele version	Known mutation**	Phenotypic effect	Interaction with other genes	OMIA ID	Reference
ASIP	agouti signaling protein	A	A	*	agouti/tabby		000201-9685	[31]
			a	2 bp deletion in exon 2	non-agouti	Epistatic to tabby modifiers (Ta, Wb)		
TYRP1	tyrosinase-related protein 1	B	B	*	black eumelanin		001249-9685	[6]
TYR	tyrosinase	C	C	*	full colour		000202-9685	[36]
			c^S	940G>A in exon 2	acromelanism			
MLPH	melanophilin	D	D	*	full colour		000031-9685	[44]
			d	83delT	dilution			
MC1R	melanocortin 1 receptor	E	E	*	black eumelanin		001199-9685	[6]
ARHGAP36	Rho GTPase activating protein 36	O	O	5.1 kb deletion within an intron	pheomelanin		001201-9685	[22, 23]
			O′ (o)	*	eumelanin			
LVRN (TAQPEP)	laeverin (transmembrane aminopeptidase Q)	Ta	Ta^M	**	mackerel pattern		001429-9685	[25]
			Ta^b	c.2522G>A	classic/blotched pattern			
KIT	receptor tyrosine kinase	W (S)	W^S	insertion of FERV1	spotting		001737-9685	[75]
			w	*	without white			
			w^g	c.1035_1036delinsCA	gloving		001580-9685	[28, 69]
			W	insertion of FERV1 long terminal repeat (LTR)	dominant white	Epistatic to all other coat colour genes	000209-9685	[75,76]
CORIN	Corin, serine peptidase	Wb	Wb	*			002159-9685	[56]
			wb^{sib}	c.2383C>T	sunshine			
			wb^{esib}	c.839G>A	extreme sunshine			

* wild–type allele; The allele symbols used by breeders are also given in brackets.

** Mutations are mainly presented based on studies of other breeds; not all variants have been molecularly confirmed in Siberian cats

Table S2. Nomenclature of exemplary coat color varieties according to FIFe and WCF standards.

Name of the variety	A	C	O	D	I
Black	aa	C/-	O′O′	D/-	ii
Blue				dd	
Red			OO	D/-	
Cream				dd	
Seal point		c^sc^s	O′O′	D/-	
Blue point				dd	
Red point			OO	D/-	
Cream point				dd	
Black tabby	A/-	C/-	O′O′	D/-	
Blue tabby				dd	
Red tabby			OO	D/-	
Cream tabby				dd	
Seal tabby point		c^sc^s	O′O′	D/-	
Blue tabby point				dd	
Red tabby point			OO	D/-	
Cream tabby point				dd	
Black smoke	aa	C/-	O′O′	D/-	I/-
Blue smoke				dd	
Red smoke			OO	D/-	
Cream smoke				dd	
Seal smoke point		c^sc^s	O′O′	D/-	
Blue smoke point				dd	
Red smoke point			OO	D/-	
Cream smoke point				dd	
Black silver tabby	A/-	C/-	O′O′	D/-	
Blue silver tabby				dd	
Red silver tabby			OO	D/-	
Cream silver tabby				dd	
Seal silver tabby point		c^sc^s	O′O′	D/-	
Blue silver tabby point				dd	
Red silver tabby point			OO	D/-	
Cream silver tabby point				dd	

Notes:

Loci B and E are monomorphic in Siberian cats, and were not included in this table, as they are in the same dominant version.

In addition all cats in this table represents recessive homozygotes genotype (w/w) in locus W. Cats with W^S/W^S, W^S/w, W^S/w^g, w^g/w^g and w^g/w genotypes will have addition "with white" the coat colour name in the end or depending on organization "with white", "bicolor", "harlequin", "van, an example seal silver tabby tortie harlequin, or cream with white. Cats with W/- will be consider white regardless of other genes. Eyes color has to be added in a case of white cats, an example, white with green eyes

Cats OO′ has an addition "tortie" to the name, an example black tortie tabby, cream tortie point with white

Tabby cats with recessive variants of Wb locus (wb^{sib}, wb^{esib}) are the sunshine cats and depending on organization "sunshine" or "golden" has to be added, an example black golden mackerel tabby

The pattern of the tabby is added before word "tabby", an example: black mackerel tabby, red classic tabby, blue spotted tabby
